# Emerging Point-of-care Technologies for Food Safety Analysis

**DOI:** 10.3390/s19040817

**Published:** 2019-02-17

**Authors:** Jane Ru Choi, Kar Wey Yong, Jean Yu Choi, Alistair C. Cowie

**Affiliations:** 1 Department of Mechanical Engineering, University of British Columbia, 2054–6250 Applied Science Lane, Vancouver, BC V6T 1Z4, Canada; 2 Centre for Blood Research, Life Sciences Centre, University of British Columbia, 2350 Health Sciences Mall, Vancouver, BC V6T 1Z3, Canada; 3 Department of Chemical & Petroleum Engineering, Schulich School of Engineering, University of Calgary, Calgary, AB T2N 1N4, Canada; 4 Faculty of Medicine, University of Dundee, Dow Street, Dundee DD1 5EH, UK; j.y.choi@dundee.ac.uk (J.Y.C.); a.c.cowie@dundee.ac.uk (A.C.C.)

**Keywords:** point-of-care devices, paper-based devices, chip-based devices, food safety analysis

## Abstract

Food safety issues have recently attracted public concern. The deleterious effects of compromised food safety on health have rendered food safety analysis an approach of paramount importance. While conventional techniques such as high-performance liquid chromatography and mass spectrometry have traditionally been utilized for the detection of food contaminants, they are relatively expensive, time-consuming and labor intensive, impeding their use for point-of-care (POC) applications. In addition, accessibility of these tests is limited in developing countries where food-related illnesses are prevalent. There is, therefore, an urgent need to develop simple and robust diagnostic POC devices. POC devices, including paper- and chip-based devices, are typically rapid, cost-effective and user-friendly, offering a tremendous potential for rapid food safety analysis at POC settings. Herein, we discuss the most recent advances in the development of emerging POC devices for food safety analysis. We first provide an overview of common food safety issues and the existing techniques for detecting food contaminants such as foodborne pathogens, chemicals, allergens, and toxins. The importance of rapid food safety analysis along with the beneficial use of miniaturized POC devices are subsequently reviewed. Finally, the existing challenges and future perspectives of developing the miniaturized POC devices for food safety monitoring are briefly discussed.

## 1. Introduction

Food safety issues have currently gained significant public health concerns. The incidence of foodborne poisoning has increased drastically [1]. According to the World Health Organization (WHO), nearly two billion people worldwide die every year from diarrheal diseases caused by bacteria, parasites and viruses, which were spread by contaminated food or water. Among them, around 30% were children under five [2]. In the United States, approximately 33 million foodborne illnesses and 9000 deaths are reported annually [3]. Foodborne illnesses are commonly caused by food contaminants (e.g., foodborne pathogens, chemicals, allergens and toxins), resulting in a wide array of symptoms from mild gastroenteritis to organ dysfunction or life-threatening syndromes [4]. These incidences have led to the efforts by the agriculture and food industry to develop analytical technologies in order to improve food safety and quality. There are several well-established food safety analysis techniques such as high performance liquid chromatography (HPLC), gas chromatography (GC), quantitative real time polymerase chain reaction (qPCR) and enzyme-linked immunosorbent assay (ELISA) [5]. However, these techniques are not only expensive, but also time-consuming and labor-intensive, making them less applicable in the rural areas or developing countries where foodborne illnesses are prevalent. To this end, there is a high demand to develop cost-effective and robust analytical devices for food safety monitoring in remote settings in order to create effective prevention and control strategies. 

With the advances in point-of-care testing (POCT), researchers have sought to develop microfluidic chip-based devices (e.g., poly(methyl methacrylate) (PMMA), polydimethylsiloxane (PDMS)-based chips) and paper-based devices (e.g., lateral flow test strips and three-dimensional paper-based microfluidic devices), which are fast gaining popularity for use in detecting food contaminants [5,6]. These emerging technologies offer numerous advantages such as being Affordable, Sensitive, Specific, User-friendly, Rapid and Robust, Equipment free, and Deliverable to end users (ASSURED), offering a high prospect as alternatives to the conventional benchtop detection technologies [7]. The detection approaches are usually based upon colorimetric, fluorescent and electrochemical detection, and the signals can be observed in a rapid and simple manner, providing effective platform for food safety monitoring [8].

While POC devices have been extensively studied, several challenges remained to be addressed before their translation into practical application. To address these formidable challenges, ongoing efforts have been devoted to achieve sensitivity improvement, quantification, multiplexing and multi-functionality in POC food safety assessment [8]. For instance, signal amplification techniques were integrated into paper- and chip-based devices to enhance their analytical sensitivity such as silver enhancement technique [9] and dual labeling technique [10]. Some devices were coupled with smartphone-based readers to achieve the quantitative detection of foodborne pathogens and chemicals [11,12]. In addition, fabricating POC devices with the ability of multiplexed detection has also attracted considerable scientific interest, which could significantly reduce assay time and cost, thereby increasing assay productivity [13]. More recently, numerous groups have developed integrated sample-to-answer devices that incorporate multiple key processing steps such as sample preparation (e.g., filtration, concentration and separation) and nucleic acid testing (NAT) steps (e.g., nucleic acid extraction and amplification) into a single device prior to target detection [14]. The advent of these technologies has remarkably enhanced the functionality of POC device to achieve more effective food safety analysis and quality control.

The escalating demand for rapid food safety analysis presents a strong need for a timely review of recent advances in the development of POC devices to investigate food safety. We first highlight the importance of rapid food safety analysis with an overview of the common food safety issues and the conventional detection technologies. The most recent development of POC devices, including paper- and chip-based devices for food safety applications, covering studies published between 2014 and 2018 are subsequently reviewed. Finally, the existing challenges and future perspectives of developing robust and portable POC devices for food safety analysis are briefly discussed. 

## 2. Importance of Rapid Point-of-Care Testing for Food Safety Monitoring

Foodborne illnesses and death are a constant threat to public health and a significant impediment to socio-economic development worldwide [15]. These incidents were usually caused by harmful food contaminants such as pathogens and chemicals. For example, an *Escherichia coli* O104:H4 outbreak in Germany attributed to contaminated fenugreek sprouts resulted in 386 cases of illness and 54 deaths in 2011 [16]. Melamine contamination of infant milk formula in 2008, which caused 2,940,000 cases of illnesses, 50,000 hospitalizations and at least 6 deaths, remains one of the largest food contamination scandals ever in China [17]. Another severe incident was associated with the illegal use of the industrial plasticizer di(2-ethylhexyl) phthalate (DEHP) as clouding agent in foods and beverages made in Taiwan, indicating improper food safety practices in the food industry [18]. These incidences have led to an increase in public awareness about food safety. Effective methods for the detection of food contaminants in foods and beverages are becoming integral to improve public health.

Conventional laboratory-based methods for the detection of food chemicals are HPLC and GC [19]. These methods require benchtop equipment systems, which are typically expensive, and the procedures are time consuming, labor intensive and usually require highly trained workers [20]. As for the detection of foodborne pathogens, culturing and plating assays are known as the gold standard [21]. However, it takes hours to days to produce the results. ELISA is able to detect targets quicker but compared to other methods, it has a lower specificity and needs multiple processing steps including rinsing and reagent addition steps, which require few hours to complete the tests [22]. Similar to ELISA, qPCR is tedious and requires numerous operation steps (i.e., nucleic acid extraction, amplification and detection) [23]. Collectively, the abovementioned conventional detection methods are high-cost and laborious, making them less applicable in resource-poor settings with limited accessibility to well-established laboratories. To overcome the problem, various emerging POC devices, such as paper- and chip-based devices, have been developed to rapidly, sensitively and specifically detect food contaminants for food safety monitoring [24]. The comparisons of existing and the emerging food safety technologies are summarized in Table 1. These emerging devices enable routine food inspection with several advantages including: (i) being cost-effective, (ii) high portability for on-site analysis, (iii) high throughput, (iv) requiring a low volume of reagents and samples, (v) having simple operation steps, and (vi) short analysis time [20,25]. These characteristics offer enormous potential for improving food safety issues, especially in the developing and underdeveloped countries, where a high incidence of foodborne illnesses is present. The details of emerging POC devices are summarized in Table 2 and will be briefly discussed in the following sections. 

## 3. Recent advances of POC Diagnostic Techniques for Food Safety Applications

### 3.1. Paper-Based Devices

Paper-based devices, particularly lateral flow test strips and microfluidic paper-based analytical devices (μPAD) are known as one of the most broadly used POC devices for food safety analysis [33]. As compared to other substrates such as silicon, glass and plastic, paper has attracted more scientific attention as it offers several advantages such as simple, affordable, biodegradable and ease-of-fabrication, modification and functionalization [100,101]. These characteristics have made it possible to achieve rapid, on-site POCT in remote settings with limited accessibility to laboratory infrastructure. The applications of paper-based devices will be briefly discussed in the following sections based on various detection methods, including colorimetric, fluorescent, chemiluminescent and other methods.

#### 3.1.1. Colorimetric Detection

Colorimetric detection represents the most common detection method in paper-based assays for food safety monitoring mainly due to its simplicity and ability to be detected by the naked eyes [102]. In fact, a numbers of lateral flow test strips have been developed for the identification of food contaminations such as foodborne pathogens, chemicals and food adulterations [103]. Generally, in lateral flow assay, the amount of targets could be estimated based upon the gold nanoparticles (AuNPs)-induced color changes. The positive result showed red signals at both control and test lines whereas the negative result showed red signal only at the control line [104,105,106,107]. With advances in paper fabrication technologies, such as printing, stacking and folding, μPAD has also been widely available for food safety applications. These devices offer many unique advantages, including the capability of storing reagents and controlling fluid transport with simple operation steps [108]. For instance, a wax-printed paper-based ELISA was developed to potentially substitute the lengthy and equipment-dependent conventional ELISA for the detection of food contaminants in POC settings [35,36]. In another study, a μPAD with well-plate design was developed for the detection of *Cronobacter* spp. [37]. The detection is achieved based on the chromogenic reaction between enzymes (i.e., a-glucosidase) specifically produced by the pathogen and enzyme substrates (i.e., 5-bromo-4-chloro-3-indolyl-α-D-glucopyranoside), producing the aglycone 5-bromo-4-chloro-3-indole that shows indigo. The colour change of paper substrate was detectable by the naked eye, indicating the presence of target pathogen. Besides pathogen detection, μPAD has also been fabricated for the colorimetric detection of food chemicals such as nitrite ions [38], benzoic acid [39] and copper ions [40] in food and water, which is simple and cost-effective, and it can be operated by the untrained users, highlighting its significant use for rapid food safety monitoring.

While many paper-based devices appear to be ubiquitous, one of the main limitations is poor sensitivity [109]. Given the fact that some existing test strips failed to detect trace amounts of contaminants in foods, new generations of lateral flow test strips have been continuously explored to improve their detection sensitivity. Recent efforts have been devoted to improve the sensitivity of lateral flow test strip for the detection of food contaminants. For example, lateral flow test strips were developed to sensitively detect *Escherichia coli (E. coli)* O157:H7 based on aptamer-mediated strand displacement amplification [41]. One aptamer was used for the enrichment of target-bound magnetic beads while the other one was used as a signal reporter, which was amplified by strand displacement amplification (SDA) and subsequently detected by a lateral flow test strip. This method allowed only the specific targets to be amplified, thereby enhancing both detection specificity and sensitivity. 

Besides that, improving detection specificity has also been one of the major challenges [33]. To achieve this, some studies have incorporated molecularly imprinted polymers (MIP) into the device to improve detection specificity [110]. MIP is generally a type of biomimetic materials that have attracted significant interest in food industry due to their favorable selectivity and sensitivity for target analytes, high physicochemical stability and long shelf life in addition to their cost-effectiveness. A recent study has demonstrated the integration of MIP into the devices to detect 17β-estradiol (17β-E2) [42]. The high efficiency and binding capacity of MIP-grafted paper-based device shows tremendous potential for practical applications.

Current works have also focused on integrating smartphone with the paper-based devices, particularly lateral flow test strips, to achieve target quantification [43]. The devices usually consist of multiple components such as robust computational processors, high quality optical sensors, wifi adaptor, rechargeable batteries and visual display along with customized applications. These efforts would significantly improve the device performance such as target quantification and signal analysis. The amount of targets can then be accurately determined by signals (e.g., colorimetric, fluorescent or luminescent signals) shown in the smartphone images. For instance, one study has demonstrated the integration of smartphone with test strip to detect alkaline phosphatase in milk [43]. Phosphotyrosine-capped AuNP was used as a signal reporter at the test zone. The images of the test zones were captured and analyzed by the smartphone without requiring any benchtop equipment, offering a good potential for in-field detection of various food analytes for rapid food safety monitoring. In addition to lateral flow test strip, some studies also combined μPAD with a smartphone for the quantification of target analytes in food. While a few studies have reported the use of smartphone in paper-based ELISA to capture the signal on paper corresponding to the amount of foodborne pathogens (e.g., *E. coli*) [11] or food chemicals (e.g., clenbuterol and nitrite, etc.) [36,44], smartphone apps have not been well-established for on-site data analysis. As a result, in most cases, external computer devices are still required for image analysis. Recent studies have been focusing on developing smartphone apps to detect food contaminants [111]. The smartphone was composed of a built in gyro-sensor and an installed software applications, which was combined with paper-based devices to achieve rapid target quantification (e.g., *E. coli*) without the requirement of any external hardware. The result showed excellent agreement with that of the conventional bacteria counting. Further investigations are required to produce reliable smartphone apps for on-site, rapid target quantification and data analysis.

The other challenges of paper-based assays in food safety applications include lack of multiplexing capability [112]. The challenges would be to simultaneously detect multiple target analytes in one or more samples using a single device while reducing the risk of cross-reactivity among target analytes. Recent studies have reported the development of a lateral flow test strip with multiple detection zones embedded with different capturing molecules to detect amplicons tagged with different antibodies [45]. For instance, a test strip coated with three different antibodies, anti-hex Ab, anti-FITC Ab, and anti-digoxin Ab was developed [45]. This device was able to detect FITC-, hex- and digoxin-tagged loop mediated isothermal amplification (LAMP) amplicons, corresponding to three main toxin genes of *Pseudomonas aeruginosa (P. aeruginosa)* (i.e., ecfX, ExoS and ExoU genes) in environmental and drinking water for potential food safety monitoring. More recently, another study has demonstrated the development of a double-strip rotary detection device integrated with two test strips to attain multiple detections of foodborne pathogens [13]. The device consists of four test lines with four different capturing antibodies to detect *E. coli* O157:H7, *Staphylococcus aureus* (*S. aureus*), *Salmonella typhimurium* (*S. typhimurium*) and *Bacillus cereus* (*B. cereus*) in the presence of specific gold-nanoparticles-conjugated antibodies (AuNP-Ab), thereby allowing multiplexed detection of target analytes. The device was able to sensitively detect bacteria in contaminated lettuce with the detection limit of approximately 1 × 10^4^ CFU/g without an external sample enrichment step.

Additionally, a great challenge remains in improving the functionality of paper-based devices for food safety analysis. Limited functionality usually refers to either the need for tedious and complex operation steps or the inability of devices to achieve on-board reagent storage or sample pretreatment prior to detection, which includes sample concentration, separation and extraction [33]. Advances in paper-based fabrication and modification technologies today have made it possible to simplify device fabrication, sample handling and processing steps as well as integrating sample pretreatment steps with minimal operation steps [113]. The challenge would be the requirement for controlling fluid transports in papers based upon the physical (structure) or chemical properties of papers. 

**Figure 1:**
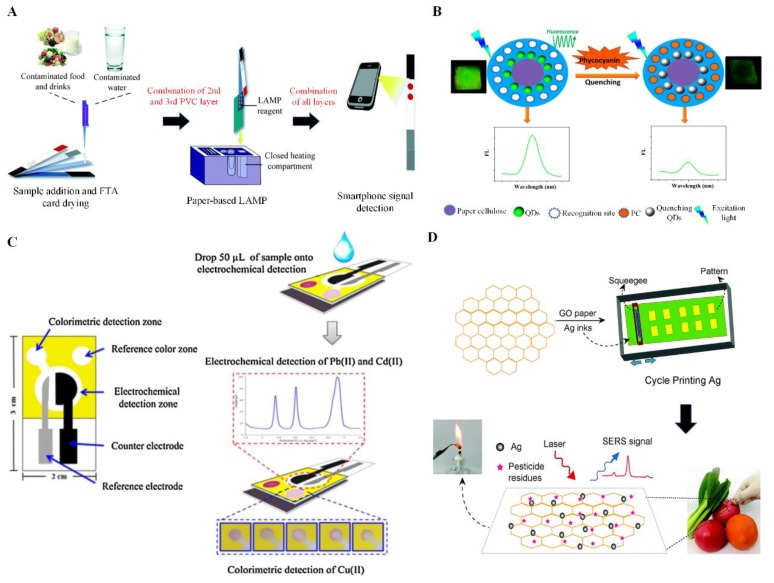
Emerging paper-based point-of-care (POC) devices for food safety analysis. (**A**) An integrated sample-to-answer paper-based colorimetric device was developed for the colorimetric detection of *E. coli* in contaminated food and drinks. Adapted with permission from [14] © Royal Society of Chemistry (2016). (**B**) A paper-based fluorescent device coupled with fluorescent quantum dots (QDs) and MIPs was introduced for the sensitive detection of phycocyanin (PC). Adapted with permission from [49] © ACS Publications (2017). (**C**) A paper-based electrochemical-colorimetric hybrid device was developed for multiplexed detection of food chemicals, including lead (II), cadmium (II) and copper (II) ions. Adapted with permission from [59] © Elsevier (2016). (**D**) A paper-based surface-enhanced Raman scattering (SERS) device was fabricated with the integration of graphene oxide (GO) and silver (Ag) nanoparticles for the detection of pesticide residues in foods. Adapted with permission from [61] © Royal Society of Chemistry (2018).

Several studies have focused on developing fully integrated paper-based sample-to-answer devices for the detection of food contaminants. For instance, a recent study has successfully orchestrated the three key steps of NAT, which involves nucleic acid extraction, LAMP and colorimetric detection into a simple four-layered integrated paper-based device [14] (Figure 1A). Flinders Technology Associates (FTA) card and glass fibers were used as substrates for nucleic acid extraction and amplification, respectively. The paper layers were initially separated by polyvinyl chloride (PVC) acting as valves to control the transport of samples from extraction zone to amplification and lateral flow zones. The integrated device could detect *E. coli* (as a model analytes) in spiked drinking water, milk and spinach with a detection limit of as low as 10–1000 CFU/mL, highlighting its potential use in food safety monitoring. To further increase the functionality of paper-based device, more recently, a fully integrated and disposable device was introduced which consists of a sponge-based reservoir for reagent storage, paper-based valve and glass fiber for paper-based DNA extraction [114], on-board battery and heater for helicase dependent amplification (HDA) and lateral flow test strip for the detection of *S. typhimurium* (as a model analyte) [46]. The device enables dried reagent storage, equipment-free amplification and rapid colorimetric detection with simple operation steps, offering a great deal of potential for food safety and quality control. Although all these efforts have successfully improved the simplicity, portability and usability of paper-based devices in food safety monitoring, the challenges of further simplifying user steps and multiplex detection should be addressed for practical applications.

Apart from NAT, numerous studies have also presented an improved functionality of paper-based device for colorimetric immunoassay or antigen detection [47]. For instance, a simple pressed paper-based device was developed to detect multiple foodborne pathogens based on the principles of antigen-antibody interactions with one-step operation. The device consists of channel partitions of lateral flow assays, which are made of nitrocellulose membrane. The desirable programming of fluid transport was achieved by pressing the nitrocellulose membrane after dipping the device into sample solutions. The colorimetric signals were developed through rehydration of AuNP-Ab with target pathogens (i.e., antigens) and the signals were amplified by rehydration of gold enhancers. This integrated device was able to simultaneously detect *E. coli* O157:H7 and *S. typhimurium* in a single step.

#### 3.1.2. Fluorescence Detection

Apart from colorimetric detection, most paper-based devices have also been widely available for fluorescent detection of food contaminants. It has been reported that fluorescent detection was able to produce a more sensitive signal than colorimetric detection. Multiple fluorescent-based lateral flow test strips have been developed to detect food contaminants. For example, one study has developed Eu (III)-doped polystyrene nanoparticle-based test strip to detect *E. coli* O157:H7 [48]. This fluorescent-based test strip was highly sensitive and specific, serving as an alternative to the conventional colloidal gold-based immunochromatographic test strip. To further improve the sensitivity, another group has combined fluorescent quantum dots (QDs) with MIPs in μPAD for specific recognition and sensitive fluorescent detection of phycocyanin in a simple and robust manner [49] (Figure 1B). QDs are typically small, fluorescent nanocrystals, which have been extensively used in device development due to their unique optical and photochemical properties and narrow emission band, enabling ultra-sensitive detection of targets for diverse applications. On the other hand, MIPs were utilized in the study due to their reusability and excellent specificity and selectivity. The device was tested with real samples of phycocyanin in water, showing a strong potential for detecting different analytes for food safety monitoring. In another study, graphene oxide (GO) was incorporated into the test strip for sensitive detection of foodborne pathogens [50]. GO is generally a two-dimensional nanomaterial with an excellent distance-dependent fluorescence quenching property, which serves as a quencher to different fluorescent dyes, enabling highly sensitive detection. Antibody decorated CdSe@ZnS QDs were printed on the test lines as capture probes and GO was added as a target-revealing agent following the sample addition. In the absence of target pathogen, the test line was efficiently quenched by the resonance energy transfer because of the close distance between Ab-QDs (donor) and GO (acceptor), leading to an “off” state. In the presence of target pathogen, the target was bound to the Ab-QDs on the test line. The test line was turned “on” after the GO was added. This is because of the failure of resonance energy transfer as the distance between donor and acceptor exceeded the distance in which resonance energy transfer is visible (>20 nm). The target was selectively bound to the test line leading to a highly sensitive and selective detection of *Salmonella* sp., achieving the detection limit of 100 CFU/mL in water and milk. The device was proved valuable as a cost-effective device for POC use.

Besides that, some studies have also showed the ability of target quantification in paper-based fluorescent devices. For instance, one study has reported the use of smartphone for fluorescent detection of *S. typhimurium* from poultry packaging solution [51]. The fluorescent signals were captured using an iPhone attached to a miniaturized fluorescent detector and the pixel intensity was analyzed using ImageJ software for target quantification. It was found that the data collected using the smartphone were in line with those of the benchtop reader system. Similarly, in another study, a smartphone was integrated with paper-based device for the detection of thiram pesticide for potential food safety control [52]. The upconversion nanoparticles were fixed onto the filter paper to create the test paper. By utilizing the 3D printing technology, the test paper, mini-laser, optical filter and mini-cavity were integrated for imaging and target quantification. In the presence of target pesticide, the blue light would be quenched through fluorescence resonance energy transfer mechanism. The fluorescent intensities could be monitored by the smartphone for quantitative analysis of thiram. This device offers new opportunities for accurate POC food safety monitoring.

In addition, numerous paper-based fluorescent devices have been introduced for multiplexed analysis. For instance, a study has developed a 10-channel up-converting phosphor technology-based lateral flow device for the simultaneous detection of multiple foodborne pathogens [53]. Ten individual strips were integrated into a single device to rapidly detect 10 pathogens: *E. coli* O157:H7, *S. paratyphi A*, *S. paratyphi B*, *S. paratyphi C*, *S. typhimurium*, *S. enteritidis*, *S. choleraesuis*, *Vibrio cholera O1*, *V. cholera O139*, and *V. parahaemolyticus* with a sensitivity of 10^4^ CFU/mL-10^5^ CFU/mL without sample enrichment. This device is highly suitable for rapid detection and surveillance of foodborne pathogens in food and water. In another study, a simple and low-cost μPAD integrated with fluorescence labeled single-stranded DNA (ssDNA) was functionalized with GO for the multiplex detection of various food contaminants, including mercury (II) ion, silver (I) ion and aminoglycoside antibiotic residues in foods [54]. Paper substrates allow the integration of ssDNA-GO through physical absorption, eradicating the need for surface treatment. For the metal ion detection, the fluorescence was off or quenched when Cy5-labeled ssDNA adsorbed on the GO surface in the absence of metal ions. In the presence of target metal ions, the ssDNA spontaneously liberate from the GO surface, resulting in the emission of fluorescence. As for the antibiotic detection, the fluorescent signal was observed when Cy5-labeled ssDNA adsorbed on the GO surface in the absence of aminoglycoside antibiotic. However, in the presence of aminoglycoside antibiotic, the binding between ssDNA with GO through amine coupling results in fluorescent quenching. Taking the benefits of both paper and GO, this integrated device enables low cost, easy fabrication, highly sensitive and selective multiplexed detection of a broad range of food contaminants. 

More recently, several groups have attempted to improve the functionality of paper-based fluorescent device for food safety monitoring. For instance, the Whiteside’s group has integrated three key steps of NAT: nucleic acid extraction, amplification and fluorescent detection into a single “paper machine” [55]. This device was able to achieve the sample-to-answer process by a simple sliding motion of the strip. It was successfully used to sensitively detect food pathogens (i.e., *E. coli*) based on fluorescent detection, demonstrating the promise their strategy holds for the detection of many types of targets for diverse applications. As fluorescent approach has proved to be more sensitive than colorimetric approach, integrating a smartphone with the sample-to-answer paper-based fluorescent device for accurate quantification coupled with multiplexing capability would pave the way for more promising food safety analysis.

#### 3.1.3. Electrochemical Detection

In addition to colorimetric and fluorescent detection, electrochemical detection has also been broadly utilized in paper-based assays for food safety applications owing to their portability, excellent sensitivity and selectivity [115]. However, traditional electrochemical device suffers from the fouling effects due to the adsorption of oxidation products on the surface of electrode. To this end, one study has developed an inexpensive and disposable paper-based electrochemical device for the detection of nitrite for food safety assessment and environmental monitoring [56]. Graphene nanosheets and AuNP were incorporated into the device due to their excellent physicochemical properties, enabling the thin layer diffusion of target analytes instead of planar diffusion at the conductive electrode. Additionally, the undesirable fouling effects could be avoided due to its disposability. As compared to the commercial glassy carbon electrodes and gold electrodes, the paper-based electrodes showed a higher sensitivity and improved electron transfer behavior for nitrite detection, paving the way for accurate food safety analysis. 

In another study, a paper-based electrochemical device was developed to detect ethanol in commercial beers [57]. Ethanol content is generally one of the major elements of beer to ensure the authenticity of the beer and hence rapid determination of the ethanol content is in high demand. To achieve this, a nanocomposite formed by carbon black and Prussian blue nanoparticles was utilized as an electrocatalyst in the device to detect the hydrogen peroxide (H_2_O_2_) generated by the enzymatic reaction between ethanol and alcohol oxidase (AOx). The developed device was able to detect ethanol up to 10 mM (0.058% vol) with a detection limit of approximately 0.52 nM (0.003% vol) in different types of beers including lager, Pilsner, weiss, and alcohol-free beer.

To achieve multiplex detection, one study has developed a μPAD with dual electrochemical and colorimetric detection was also developed for simultaneously detection of lead (II), cadmium (II) and copper (II) ions in a single sample [59] (Figure 1C). The μPAD was divided into two parts. The first part involves electrochemical detection of the lead (II) and cadmium (II) ions by using a bismuth-modified, boron-doped diamond electrode as a working electrode, whereas the second part involves the colorimetric detection of copper (II) ion using the catalytic etching of silver nanoplates by thiosulfate. The change of color from violet to colorless indicates the presence of copper (II) ion. The proposed device was able to achieve the detection limit of 0.1 ng/mL for lead (II) and cadmium (II) ions and 5 ng/ml for copper (II) ion, which could be extremely useful for rapid POC water analysis.

Furthermore, to improve functionality and simplicity of the electrochemical assay, one group has developed an innovative paper-based electrochemical device for impedimetric detection of pathogens [58]. The device was fabricated by screen-printing carbon electrode onto a hydrophobic paper without the use of capturing molecules such as antibodies, which significantly reduces the fabrication cost and improves simplicity. Concanavalin A was selected as the recognition element due to its high selectivity towards monosaccharides or oligosaccharides on bacteria. The device could sensitively detect bacteria from sewage sludge, achieving a detection limit of approximately 2 × 10^3^ CFU/mL, which was lower than previously reported impedimetric devices, showing great promise in water and food safety assessments.

#### 3.1.4. Other Detection Methods

Apart from paper-based colorimetric, fluorescent and electrochemical devices, other devices such as paper-based chemiluminescent devices and paper-based surface-enhanced Raman scattering (SERS) devices have also been developed for the detection of food contaminants. For example, Z-folding controlled liquid handling μPAD was developed for the chemiluminescent detection of foodborne pathogen via adenosine triphosphate (ATP) quantification [60]. The device was fabricated by wax printing to create reagent and detection zones. For sample testing, sample solution was first added to the reagent zone, which consisted of immobilized ATP aptamer and horseradish peroxidase (HRP) tagged DNA. The binding of target would result in the release of HRP tagged DNA, which would flow to the detection zone that composed of 3-amino-9-ethylcarbazole (AEC) through a simple folding of paper. Lastly, H_2_O_2_ would be added to the detection zone to visualize the color changes. In the presence of target, the color of detection zone would be changed from light yellow to dark red corresponding to the concentration of target. The proposed device was used for the detection of *Salmonella sp*., suggesting the potential of POC food monitoring.

In addition, SERS have also been coupled with paper-based device for the detection of food contaminants. This technology has been widely investigated owing to its significant enhancement of Raman scattering signals, which usually involves the use of metal substrates such as gold or silver nanoparticles (AgNPs) [116]. SERS collects vibrational signals associated with various functional groups of target molecules, leading to a sensitive and specific detection [117]. In a recent study, a paper-based SERS device was introduced for the detection of pesticide residues, including thiram, thiabendazole and methyl parathion in apples and green vegetables [61]. This device was fabricated by incorporating AgNPs and GO onto a cellulose paper using screen-printing technique for enrichment and SERS detection of pesticide residues (Figure 1D). The proposed device showed high enrichment ability, allowing the pesticide residues to be adsorbed on GO via π-π stacking and electrostatic interactions. Additionally, it exhibits excellent SERS activity, showing a great promise for POC environmental analysis and food safety applications.

In another study, paper-based SERS device was developed by hydrophobic modification of filter paper with high sensitivity and reproducibility [62]. The filter paper was subjected to the hydrophobic treatment of alkyl ketene dimer to prevent the absorption of AgNP and sample solutions by the paper. Unlike conventional filter papers, the hydrophobic modified filter paper produced more SERS hot-spots consisted of AgNP clusters on the paper surface, resulting in a more sensitive detection of pesticides. Pesticides such as thiram and ferbam could be detected at the nanomolar level using the proposed device, demonstrating its high applicability to detect pesticides in a cost-effective manner.

### 3.2. Chip-Based Devices

Apart from paper-based devices, scientists have also sought to develop chip-based devices for POC food safety analysis, which are mainly made of PDMS or PMMA [20]. These devices enable precise control and manipulation of small amount of samples to achieve automation and high throughput analysis. The development of chips has eliminated the requirement for external instruments and components for operations such as power supplies, benchtop detectors and computers, hence reducing the overall size and cost of the devices [118]. Similar to paper-based devices, most existing chip-based devices serve as a platform for the detection of food contaminants based on colorimetric, fluorescent and electrochemical approaches.

#### 3.2.1. Colorimetric Detection 

For colorimetric detection, one of the challenges that are currently being addressed is poor sensitivity. To address this challenge, a recent study has incorporated silver enhancement technique into a chip-based ELISA to sensitively detect aflatoxin B1 (AFB1) [9]. The sample solution was initially mixed with biotinylated anti-AFB1 Ab, followed by the addition of AuNP-streptavidin conjugates. Silver stain was subsequently added into the solution to amplify the colorimetric signal of AuNP-streptavidin conjugates. As a result, this chip-based ELISA was able to sensitively detect AFB1 in corn, achieving the detection limit of 3 ppb, which was consistent with the result produced by conventional ELISA.

Besides sensitivity enhancement, several chip-based colorimetric device have been developed to achieve simple and rapid target quantification. For example, a chip-based colorimetric device was incorporated with a portable detector for quantifying the colorimetric signals without relying on a benchtop equipment. Further, some studies have integrated a handheld custom-made colorimetric reader with chip-based devices to detect the colorimetric signal and convert the light intensity to an electrical signal for quantitative analysis. For instance, one device was able to sensitively detect food allergens gluten and Ara h1 in wheat and peanut, respectively [63], whereas the other device was able to sensitively detect heavy metal ions such as lead (II) and aluminium (III) ions in water [64]. Besides that, a smartphone can be used to directly quantify the color intensity of sample. For instance, in one study, this approach was used to determine concentration of malathion (an organophosphate pesticide) in apple and tetrabromodiphenyl ether (a harmful chemical) in water [65,66]. However, these devices require a computer for data analysis. To improve the applicability of chip-based devices at resource limited settings, smartphone apps have been developed and integrated into the chip for on-site target quantification. For instance, a smartphone with a custom-designed imaging app named “AFBIDET”, was integrated into a chip-based ELISA to quantify AFB1 in corn without the need of any external hardware [9].

**Figure 2:**
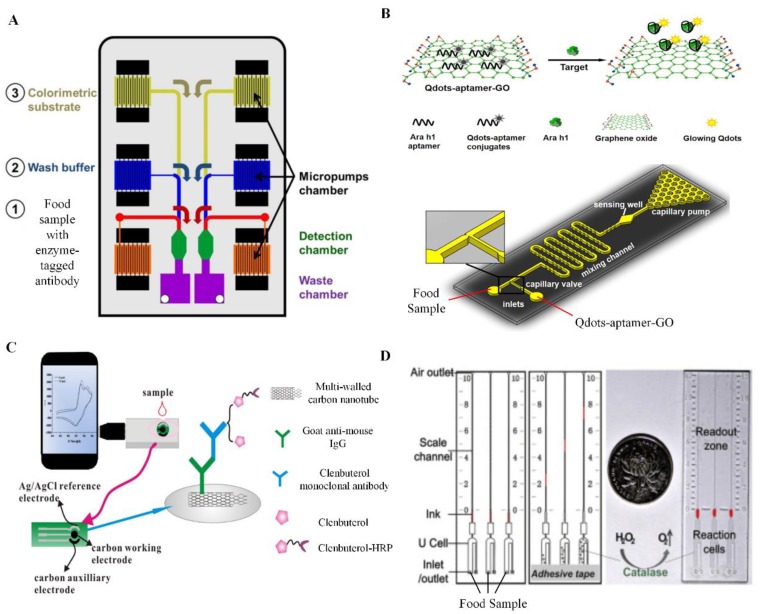
Emerging chip-based point-of-care (POC) devices for food safety analysis. (**A**) An automated chip-based enzyme-linked immunosorbent assay (ELISA) was developed for colorimetric detection of tetrabromodiphenyl ether in water. Adapted with permission from [66] © AIP Publishing (2014). (**B**) A chip-based fluorescent device coupled with quantum dots (Qdots), aptamer and graphene oxide (GO) was developed for sensitive detection of food allergen Ara h1 in biscuit. Adapted with permission from [68] © Elsevier (2016). (**C**) A chip-based electrochemical device functionalized with antibody immobilized multi-walled carbon nanotube was developed for rapid detection of clenbuterol in water. Adapted with permission from [77] © Elsevier (2016). (**D**) A volumetric chip was introduced for the detection of bovine catalase in milk without the requirement of any external detector. Adapted with permission from [83] © Elsevier (2016). Ag: silver; AgCl: silver chloride; IgG: immunoglobulin G; HRP: horseradish peroxidase; H_2_O_2_: hydrogen peroxide; O_2_: oxygen.

Similar to paper-based devices, multiplexing capability and multi-functionality of chip-based colorimetric devices remains challenging today. To achieve multiplexed detection, some existing chips were integrated with multiple microchannels or microwells that can be coated with different capturing antibodies [9,64,119]. As an example, a chip-based ELISA was developed for simultaneous colorimetric detection of different types of mycotoxin such as deoxynivalenol, aflatoxin B1 and ochratoxin A in corn [119]. To improve the device functionality, a recent study has demonstrated the utilization of a smartphone to control pumps being placed inside a microfluidic chip [66] (Figure 2A). This device was able to achieve automated ELISA, which simplified several key steps, including: (i) the mixing of target analyte and its enzyme-tagged antibodies, (ii) rinsing with wash buffer and (iii) mixing with colorimetric substrate in a detection chamber in a sequential manner, which greatly reduced operation steps. The operation power was continuously supplied by the smartphone to perform entire tests without relying on electricity. The device was able to detect tetrabromodiphenyl ether in the concentration range of 10^−3^ to 10^4^ μg/L, which was comparable to that of conventional ELISA. In short, achieving both multiplexing and multi-functionality in a single device are instrumental in POC food safety analysis to significantly reduce assay cost and time. 

#### 3.2.2. Fluorescent Detection

In addition to colorimetric devices, chip-based fluorescent devices have been widely available for POC food safety analysis due to their superior detection sensitivity and specificity [120]. Numerous efforts have been made to further enhance the sensitivity of chip-based fluorescent devices. For instance, one study has developed a chip-paper hybrid device for sensitive detection of foodborne pathogens such as *S. aureus* and *V. parahaemolyticus* [10]. This study suggested using the dual labeling of LAMP products to clearly distinguish the weak positive LAMP reaction from the negative LAMP reaction [10]. Instead of using only a single dye, a mixed-dye containing SYBR Green and hydroxyl naphthol blue was used to stain the LAMP products to improve the detection sensitivity and specificity of the existing LAMP technology [121]. Moreover, sensitivity of chip-based fluorescent devices could also be enhanced by replacing conventional organic fluorescent dyes with QD. As mentioned earlier, QD have several advantages, including high resistance to chemical degradation and high photostability. To prove the role of QD in sensitivity enhancement, a study has utilized a detection antibody tagged with QD to sensitively detect *S. typhimurium* in chicken extract within a microfluidic chip [67], whereas another study has used an aptamer conjugated with QD to sensitively detect food allergen Ara h1 in biscuit [68] (Figure 2B).

One of the limitations of chip-based fluorescent device is equipment-dependent quantification, which is unsuitable to be performed in resource-limited settings where laboratory facilities are unavailable [68,69,70]. To achieve target quantification without relying on benchtop equipment, a chip-based fluorescent device was incorporated into a portable fluorescent detector for accurately quantifying the fluorescent signal. Some studies have developed a handheld custom-made fluorometer to measure fluorescent signal and converted the fluorescent intensity to electrical signal, which requires a computer for data analysis [67,68]. To improve applicability of a chip-based fluorescent device at resource limited settings, similar to the aforementioned devices, it is essential to include a smartphone and its app for on-site data analysis. One recent study has integrated a smartphone with a custom-designed app, called “Spot-An-Array” into a protein microarray chip-based fluorescent device for on-site quantification of anti-recombinant bovine somatropin antibody in milk without the need of a benchtop instrument [72].

In addition to quantification, recent efforts also involve achieving multiplexed detection and improving functionality of chip-based fluorescent devices. To achieve multiplexed detection, a microfluidic chip was integrated with multiple reaction chambers accommodating different sets of primers for detection of various foodborne pathogens, including *E. coli*, *Proteus hauseri*, *V. parahaemolyticus* and *S. enterica,* via LAMP [70]. The limit of detection for each pathogen was as low as 3 copies/μL. To improve device functionality, some studies have integrated a sample concentration technique into the chip to concentrate target from food sample for efficient downstream fluorescent detection. For instance, immunomagnetic separation approach was integrated into a chip-based fluorescent device to separate *Salmonella* sp. and *Listeria monocytogenes* from various sources of meat sample such as pork, chicken and beef prior to detection, which significantly reduced user steps [67,69,72]. Additionally, some devices have achieved on-board reagent storage, which have also simplified operation steps. For example, Chen et al. [70] have developed a microfluidic chip that consisted of reaction chambers accommodating all LAMP reaction components embedded in an agarose gel, which allows long-term on-board sample storage for the detection of foodborne pathogens. However, in this study, DNA extraction process was performed off-chip. To further improve the functionality, Sun et al. [72] have invented a microfluidic chip with fully integrated sample preparation and all key steps of NAT (i.e., DNA extraction, LAMP and fluorescent detection) to rapidly detect *Salmonella* sp. in pork. This chip has a great potential to serve as a sample-to-answer POC platform for the real-life detection of foodborne pathogens. 

#### 3.2.3. Electrochemical Detection

Chip-based electrochemical devices have been widely developed for the detection of food contaminants, especially foodborne pathogens, due to their rapidity and high detection sensitivity. With advances in fabrication techniques of microelectronics, electrochemical assays can be readily integrated into one simple microfluidic chip with minimal operation steps at a minimal cost [122]. Chip-based electrochemical chips generally rely on attachment of labels (e.g., antibodies, metal particles and conductive polymers) or target bacterial cells (label-free) onto the electrode surface to induce measurable changes in electrical parameters such as impedance, current or potential [122,123]. For example, a label-free positive dielectrophoretic microfluidic chip was developed for safety assessment of drinking water [73]. This chip consists of a cell-focusing electrode that sorted bacteria cells (e.g., *E. coli*) out from water and a sensing electrode for capture and detection of the bacteria. The resulting impedance changes were used to enumerate the bacteria. It was found that *E. coli* at 300 CFU/mL could be rapidly detected within 1 min [73]. However, a label-free chip-based electrochemical device is prone to non-specific attachment of non-target bacteria onto the electrode surface, which could generate a false positive result. To improve the detection sensitivity and specificity, labels were used to specifically tag the target food contaminant prior to electrochemical detection. For instance, a nanoporous alumina membrane functionalized with antibodies targeted *E. coli* O157:H7 was used to specifically capture and detect the bacteria within a microfluidic chip with negligible cross-binding of non-target bacteria [74]. This approach has improved the sensitivity of impedance device for the detection of *E. coli* O157:H7, achieving a relatively low limit of detection (i.e., 100 CFU/mL). 

Quantification of food contaminants using a chip-based electrochemical device has been relying on an electrochemical analyzer such as impedance analyzer, ammeter or potentiometer [73,74,75,76]. To this end, a smartphone was integrated into the chip to quantify targets and improve applicability of a chip-based electrochemical device in resource-limited settings. For example, a smartphone with a custom-designed app was integrated into a screen-printed electrode chip functionalized with antibody for on-site rapid quantification of clenbuterol [77] (Figure 2C). The entire detection process was completed within 6 min. 

Furthermore, multiplexed detection of food contaminants is also essential in POC food safety analysis. Some studies have reported that multiple foodborne pathogens, such as *E. coli* O157:H7 and *S. aureus*, can be simultaneously detected and quantified using a single chip-based electrochemical device [74,78]. Also, similar to chip-based colorimetric and fluorescent device, one major challenge of a chip-based electrochemical device in food safety analysis is limited functionality. The performance of device could be greatly affected by the content (chemicals, carbohydrates, fat and protein), physical state (gel, liquid and solid) and viscosity of food. To address this issue, it was suggested to incorporate sample separation technique into the chips to concentrate the targets prior to electrochemical detection. For instance, magnetic nanoparticles were first tagged with antibodies targeted *E. coli* O157:H7 to concentrate the bacteria from food (e.g., milk) using a magnet followed by conjugation with AuNP modified with urease and aptamers against the bacteria [75]. These bacterial complexes were used as catalyst for hydrolysis of urea into ammonium carbonate, which were loaded into a microfluidic chip for impedance measurement. This immunomagnetic separation approach was shown to further improve the sensitivity of impedance device for the detection of *E. coli* O157:H7, achieving an extremely low limit of detection (12 CFU/mL). Another study has also used immunomagnetic separation approach to concentrate *S. typhimurium* in milk [76]. *S. typhimurium* were conjugated with AuNP and loaded into a microfluidic chip to measure electric current in order to determine bacterial concentration. However, a common limitation of both studies was the requirement of off-chip complicated sample concentration technique. Therefore, future work should involve integrating a simple sample concentration technique into the chip-based electrochemical device to enhance its applicability at POC settings. 

#### 3.2.4. Other Detection Methods

Besides colorimetric, fluorescent and electrochemical detection methods, other detection methods, such as surface plasmon resonance (SPR), gas pressure-induced ink bar advancement and turbidity, were also introduced in microfluidic chips for food safety applications. SPR has been broadly used with microfluidic chip. This technology has exploited special electro-magnetic waves to probe interactions between the analyte and a biomolecular recognition element immobilized on the SPR device surface. Several chip-based SPR devices have been developed to detect food contaminants such as mycotoxin and foodborne pathogens [79,80,81,82]. These chips were coated with a gold layer functionalized with either antibody or aptamer for plasmonic sensing. For example, an aptamer immobilized SPR chip was used to sensitively detect ochratoxin A in wine and peanut oil [79]. A major challenge of using SPR chip is fouling from food matrices on the sensing surface of SPR chip that would potentially cause a false positive signal. To circumvent this challenge, one study has integrated poly(carboxybetaine acrylamide) brushes into a SPR chip to improve its resistance to fouling from cucumber and hamburger samples [80]. As a result, *Salmonella* sp. and *E. coli* O157:H7 can be detected with high sensitivity and specificity. However, target quantification of most chip-based SPR devices has relied heavily on benchtop spectrophotometers. To improve applicability of these devices at remote settings, it is essential to incorporate a portable spectrophotometer for measuring the plasmonic signal. Such a portable integrated plasmonic platform was developed for the detection and quantification of *E. coli* and *S. aureus* [81]. Moreover, some SPR chips contain multiple microchannel or microspots that can be coated with different aptamers or capturing antibodies for multiplex detection of food contaminants [80,81,82]. With the advances in smartphone technologies, smartphone-based SPR sensing is expected to be expanding rapidly for POC food safety analysis. 

Volumetric meter chip (V-chip), which is based on gas pressure-induced ink bar advancement detection approach has currently attracted significant interest in POC food safety analysis due to its capability of on-chip target quantification without relying on any external detectors. Target quantification can be simply achieved by measuring the visualized distance change of an indicator, such as ink, in the chip [124]. One study has developed a V-chip for detection of bovine catalase in milk for food safety analysis [83] (Figure 2D). This chip contains H_2_O_2_ that can react with bovine catalase to generate oxygen (O_2_) gas, which increases pressure in a microchannel, resulting in ink bar advancement. The distance of the ink bar advancement can be measured by the scale engraved on the chip without the need of an optical detector. It was found that the higher the concentration of bovine catalase, the longer the distance of ink bar advancement. However, the V-chip has limited analytical specificity given that H_2_O_2_ can react with many catalysts to produce O_2_ gas. To improve its specificity, another study has combined a V-chip with a target-responsive hydrogel containing a specific aptamer against the target and platinum nanoparticles for food safety analysis [84] This chip was used to detect AFB1 in beer. In the presence of AFB1, the AFB1 bound to its aptamer in a polyacrylamide hydrogel, causing disruption of the hydrogel and release of platinum nanoparticles. These nanoparticles subsequently catalyzed the decomposition of H_2_O_2_ in the chip to produce O_2_ gas, resulting in significant ink bar advancement. Besides simple target quantification, these two V-chips have multiplexing capabilities as they contain multiple microchannels for multiplexed detection of food contaminants [83,84]. 

Apart from that, turbidity detection method can be integrated into a chip-based device for food safety analysis. For instance, one study has used a chip-based LAMP followed by turbidity measurement to quantify *E. coli* O157:H7 gene for detection of *E. coli* O157:H7 in food samples such as apple juice and milk [85]. Anti-*E. coli* O157:H7 functionalized carbon nanotube was integrated into the chip to concentrate *E. coli* O157:H7 prior to detection. The turbidity of LAMP reaction solution was measured with a portable fiber optic detector. It was found that the turbidity increases linearly with the concentration of *E. coli* O157:H7. However, this chip has limited functionality as sample separation and DNA extraction were performed off-chip and a computer is required for data analysis. Improving its functionality would offer new opportunities for real world applications. 

### 3.3. Other POC Devices 

In addition to paper- and chip-based POC devices, other devices like nanomaterial-, thread-, cuvette-, tube-, disc-, glass slide- and well plate-based devices have also been developed for POC food safety analysis [86,88,89,92,94,95,97]. Specifically, these devices were introduced to significantly improve the sensitivity and functionality of both paper- and chip-based devices, providing more promising options for real-world applications.

#### 3.3.1. Colorimetric Detection

**Figure 3:**
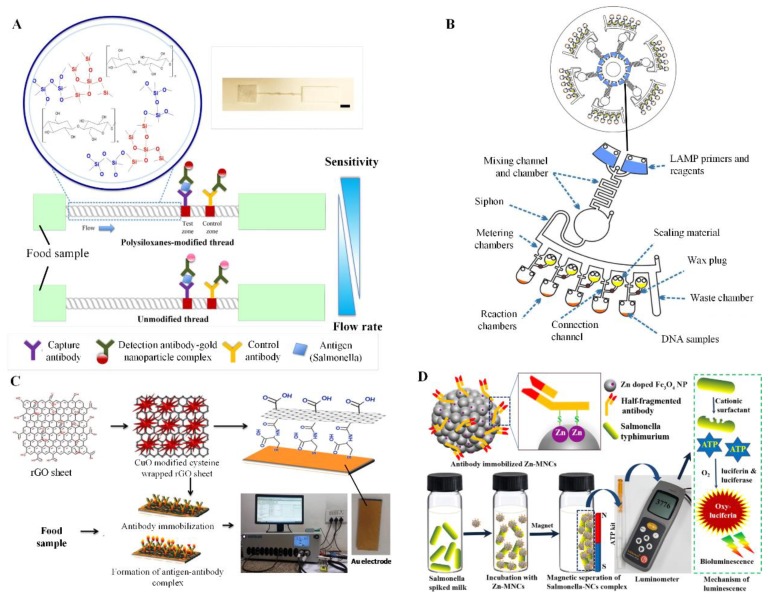
Other emerging point-of-care (POC) devices for food safety analysis. (**A**) A thread-based colorimetric device was developed for sensitive detection of *Salmonella enterica* in food samples such as milk, orange juice and lettuce. Adapted with permission from [86] © Elsevier (2018). (**B**) A disc-based loop-mediated isothermal amplification (LAMP) was developed for multiplexed fluorescent detection of foodborne pathogens, *Escherichia coli*, *Salmonella* sp. and *Vibrio cholera*, in chicken meat. Adapted with permission from [94] © Elsevier (2018). (**C**) A gold (Au) electrode coated with antibody immobilized graphene-wrapped copper oxide-cysteine hierarchical structure was introduced for sensitive electrochemical detection of *Escherichia coli* O157:H7 in food samples such as water, fruit juice and milk. Adapted with permission from [97] © Elsevier (2017). (**D**) A nanomaterial-based chemiluminescent device was developed for sensitive detection of *Salmonella typhimurium* in milk. Adapted with permission from [99] © Elsevier (2017). rGO: reduced graphene oxide; CuO: copper oxide; Zn-MNCs: Zinc-doped magnetic nanoclusters; Fe_3_O_4_ NP: Iron oxide nanoparticle; ATP: adenosine triphosphate; O_2_: oxygen.

Similar to paper- and chip-based devices, improving detection sensitivity is also one of the approaches in alternative devices. For example, a recent study has incorporated silver enhancement technique into a well plate-based ELISA to enhance its detection sensitivity [92]. Silver enhancement solution was added into the sample solution to amplify colorimetric signal of AgNP-Ab for the detection of target analytes. As a result, this device was able to sensitively detect antibiotic residues such as tetracyclines and quinolones in milk. Another study has incorporated polysiloxanes into a thread-based colorimetric device for the enhancement of detection sensitivity [86] (Figure 3A). Polysiloxanes allow desirable fluidic delay that increases interactions between targets and AuNP-Ab, producing more complexes, which were captured by the capturing antibody at the detection zone or knot. With the optimum conditions, this polysiloxanes-modified thread-based device was 10-fold more sensitive than the unmodified device in colorimetric detection of *S. enterica* in food samples such as milk, orange juice and lettuce. The sample volume required by the thread-based device (~20 µL) for testing was lower than that of traditional test strip (~50 µL), offering great potential to substitute the traditional test strip for POC applications.

Besides sensitivity enhancement, on-site target quantification has also been achieved. For example, some studies have integrated a smartphone with a custom-designed or commercial available smartphone app into cuvette-, tube- or well plate-based colorimetric devices for on-site quantification of fluoride [88,91] or mercury (II) ion [89,90] in water or marine toxins [93] in shellfish. Moreover, with the integration of a Google Map app, a spatiotemporal mapping of food contamination can be generated for food safety monitoring. For example, a cuvette-based colorimetric device integrated with a smartphone was used to produce a spatiotemporal mapping of mercury contamination in California by testing water samples collected from 50 different locations, suggesting its potential for real world applications [90]. 

In addition to quantification, it is also essential to achieve multiplexing and multi-functionality in other POC colorimetric devices. To achieve multiplexing, one well plate-based ELISA was used to simultaneously detect tetracyclines and quinolones in milk [92], whereas another well plate-based ELISA was used to simultaneously detect two types of marine toxins such as saxitoxin and okadaic acid in shellfish [93]. These devices can be integrated with a smartphone with its app for on-site target quantification and data analysis. To achieve multi-functionality, some colorimetric devices have achieved on-board reagent storage and sample-to-answer capabilities for detection of food contaminants [86,88]. For instance, one study has stored AuNP-Ab and capturing antibody on the sample pad and knot, respectively, in a thread-based device for the detection of *S. enterica* in various food samples [86]. Following the addition of the sample solution into the sample pad, the target was bound to the AuNP-Ab. The resulting target-AuNP-Ab complexes were in turn transported to the detection zone on the thread by capillary action to interact with the capturing antibody, generating red signal for quantification of *S. enterica*. The operation steps were significantly reduced in both above-mentioned devices, making them a great sample-to-answer colorimetric detection platform for POC food safety analysis. 

#### 3.3.2. Fluorescent Detection

Nanomaterials such as graphene has been used as a substrate to enhance detection sensitivity of fluorescent assays based on fluorescence quenching. One study has developed a graphene oxide-based device for sensitive fluorescent detection of *S. typhimurium* in milk sample [96]. To achieve on-site quantification, a smartphone with a custom-designed app named “GotMilk” was integrated into a glass slide-based fluorescent device for on-site quantification of anti-recombinant bovine somatropin antibody in milk [95]. Another study has integrated a smartphone into a simple disc-based LAMP for on-site fluorescent quantification of foodborne pathogens in chicken meat [94] (Figure 3B). This device can simultaneously detect three types of foodborne pathogens, including *E. coli*, *Salmonella* sp. and *V. cholera*, achieving a detection limit of 0.03 µg/µL for each pathogens. Moreover, the device enables on-board storage of LAMP reaction components, and the operations from reagent mixing to signal detection can be simply controlled by adjusting the disc rotational speed, which greatly reduced user steps. 

#### 3.3.3. Electrochemical Detection

Besides fluorescent detection, graphene has also gained interest in electrochemical sensing due to its excellent electrical conductivity and high surface area, which dramatically improved the detection sensitivity [125]. For example, one study has developed an impedance device based on graphene-wrapped copper oxide-cysteine hierarchical structure to detect *E. coli* O157: H7 in food samples such as water, fruit juice and milk, achieving the detection limit of 3.8 CFU/mL [97] (Figure 3C). In addition, a thread-based electrochemical device was developed to sensitively detect phenol in water [87]. The thread supported transportation of sample to the electrodes functionalized with tyrosinase enzyme for electrochemical detection, which has significantly reduced the operation steps. Phenol was converted to o-quinone and the resulting electrochemical signal was quantified via voltammetry to achieve accurate quantification of phenol. However, both electrochemical POC devices have some shortcomings. First, a smartphone app was not integrated into these devices for on-site data analysis. Second, the device was not able to process multiple samples simultaneously. Addressing these challenges would allow more promising food safety applications in the future. 

#### 3.3.4. Other Detection Methods

Other detection methods such as gas-pressure induced ink bar advancement and chemiluminescence have been introduced in other POC devices for food safety analysis. One study has tagged foodborne pathogens (e.g., *S. enteriditis* and *E. coli* O157:H7) with MnO_2_ nanoflowers (nanomaterial) that act as a catalyst for decomposition of H_2_O_2_ into O_2_, resulting in ink bar advancement in a disposable chamber [98]. The distance of ink bar advancement was measured by a ruler to determine the concentration of target pathogens. The MnO_2_ nanoflowers can be fabricated with a simple and environmental friendly approach at a minimal cost. However, this device has some drawbacks as compared to the previously mentioned V-chip. The device failed to achieve rapid target quantification as a ruler is required to measure the distance of ink bar advancement. It also lacks on-board reagent storage and multiplexing capabilities, which require further improvement. In another study, a nanomaterial-based chemiluminescent device was developed to determine the concentration of *S. typhimurium* in milk [99] (Figure 3D). *S. typhimurium* in milk was first tagged with anti-*Salmonella* functionalized zinc-doped magnetic nanoclusters followed by target concentration using a magnet. The ATP was subsequently extracted from the bacteria, which in turn interacted with luciferin to generate luminescent signal. A portable ATP luminometer was used to measure the luminescent signal, which shows great promise for POC applications in remote settings. In the future, simplifying operation steps would add more practical value to the device. 

## 4. Conclusions and Future Perspectives

This review article discusses the overview state-of-art research in POC technologies for food safety monitoring. The rapidity, simplicity, cost-effectiveness and portability of POC devices play a key role in a broad range of applications. Recent advances in the field have made it possible to achieve sensitive and specific detection of food contaminants. Furthermore, quantification could also be achieved by using a simple smartphone app, enabling signal analysis to be performed by untrained users without the requirement of benchtop equipment. More recently, several other materials beyond paper such as textile, nanomaterials or carbons were explored in POC applications to improve device sensitivity, simplicity and functionality [86,126]. To further improve simplicity and functionality, some studies have also attempted to integrate sample-to-answer process into a single device [14]. This robust device shows great promise for possible development into a stand-alone device for the detection of food contaminants outside the laboratory, especially in the developing countries.

Even though significant efforts have been devoted in this emerging field, there are several challenges that require attention. Future goals should focus on further simplifying the user steps by creating an automated fluidic delivery on chip as well as incorporating multiple steps into a single device (e.g., nucleic acid extraction, amplification and detection) in a simple and cost-effective manner [8]. The ability of preserving all reagents on board is crucial to eradicate the need for laboratory storage unit and the capability of multiplexing could significantly improve the assay productivity [24]. Further, more studies should focus on developing more robust smartphone apps that allow on-site analysis while providing swift transfer and data storage to keep track of records. Given the fact that wireless network supply is limited in most resource-poor settings, the device should also be supported by asynchronous data transmission. The integration of alternative power sources such as battery or solar power would remarkably improve the performance of device especially in rural areas with limited power supply [127]. In addition, integrating all key processing steps (e.g., sample collection, sample preparation and detection) into a single fully integrated device remains challenging today. Paper-based devices, in particular, could potentially achieve automated sequential fluid delivery and significantly simplified operation steps as they allow liquid transport by capillary forces [128]. Future works should focus on minimizing user steps by creating automated fluidic delivery to achieve sample-to-answer process in a simple manner. 

Novel nanomaterials have been widely explored in the field of sensing and food safety applications. The benefits of using carbon-based nanomaterials (e.g., graphene and GO), noble metal nanoparticles (e.g., AuNP and AgNP) and MIPs have also been frequently reported [54,129], especially the capabilities of producing enormous signal enhancement and amplification with high selectivity. Exploring new nanomaterials such as black phosphorus would be beneficial due to their fascinating properties such as direct bandgap, strong structural and functional anisotropy, high conductivity and electron transfer capacity [130], which could significantly improve the detection sensitivity. In fact, better understanding of the fundamentals of these materials in terms of chemical, structural and physical properties would allow engineering of these materials to produce more biocompatible substrates. The stability of these materials should also be tested to ensure its robustness and reliability for real applications.

In short, emerging POC technologies will be capable of offering robust, portable, easy-to-use, cost-effective, sensitive and specific sample-to-answer devices with automation and multiplexing capabilities. With these devices, food contaminants such as toxic chemicals, pesticides and infectious agents could be swiftly identified and quantified, mitigating foodborne illness outbreaks. 

## Figures and Tables

**Table 1:** Comparison of conventional and emerging food testing technologies.

Diagnostic test		Types of Targets	Time	Cost ($)	Diagnostic Specificity (%)	Diagnostic Sensitivity (%)	Expertise Required	Special instrument Required	Ref.
**Conventional Methods**	Bacteria culture	pathogen	~1–2 days	3–6	100	100	Yes	Yes	[26]
	ELISA	pathogen	~6 h	10	70–90	61–99	Yes	Yes	[27,28]
	qPCR	pathogen	~4 h	20	100	80–100	Yes	Yes	[29]
	GC	chemical	~30 min	20–30	95	99	Yes	Yes	[30]
	HPLC	chemical	~30 min	20–30	95	99	Yes	Yes	[31,32]
**Emerging Technologies**	POC devices	pathogen and chemical	~20–30 min	2	100	80–100	No	No	[33,34]

Abbreviation: ELISA: enzyme-linked immunosorbent assay; qPCR: quantitative polymerase chain reaction; HPLC: high performance liquid chromatography; GC: gas chromatography; POC: point-of-care.

**Table 2:** Emerging point-of-care (POC) devices for food safety analysis.

Emerging POC Devices	Test Principle	Target Analytes	Sample Type	Sample Pretreatment	Limit of Detection	Assay Time	Ref
Paper-based devices	Colorimetric	*Escherichia coli* O157:H7	Chinese cabbage	External steps of grinding and filtration	10,000 CFU/mL	~ 1 h	[35]
	Colorimetric	Clenbuterol	Milk	-	0.2 ppb	1 h	[36]
	Colorimetric	*Cronobacter* spp.	Water	External step of sample enrichment	10 CFU/cm^2^	1 h	[37]
	Colorimetric	Nitrite ion	Water	External step of filtration	0.5 nmol/L	5 min	[38]
	Colorimetric	Benzoic acid	Water	-	500 ppm	1 h	[39]
	Colorimetric	Copper ions	Water, tomato juices	External step of filtration	0.3 ng/mL	2 min	[40]
	Colorimetric	*E. coli* O157:H7	Phosphate buffered saline, milk, water and apple juice	-	10 CFU/mL	35 min	[41]
	Colorimetric	17β-estradiol	Milk	External step of target separation	0.25 μg/L	10 min	[42]
	Colorimetric	Alkaline phosphatase	Milk	External step of target separation	0.1 U/L	10 min	[43]
	Colorimetric	Nitrite	Water	External step of filtration	73 ng/mL	15 min	[44]
	Colorimetric	*Pseudomonas aeruginosa*	Water	-	20 CFU/mL	50 min	[45]
	Colorimetric	*E. coli*	Water, milk, spinach	External grinding and filtration steps for spinach On-board DNA extraction and amplification	10–1000 CFU /mL	1 h	[14]
	Colorimetric	*Salmonella typhimurium*	Milk, juice	On-board DNA extraction and amplification	100–1000 CFU /mL	1 h	[46]
	Colorimetric	i) *E. coli* O157:H7 ii) *S. typhimurium*	Phosphate buffered saline	-	i) 10^5^ CFU /mL ii) 10^6^ CFU /mL	10 min	[47]
	Fluorescence	*E. coli* O157:H7	Milk	-	~100 CFU/mL	30 min	[48]
	Fluorescence	Phycocyanin	Water	External step of filtration	2 mg/L	30 min	[49]
	Fluorescence	*E. coli*	Water, milk	-	10–1000 CFU /mL	1.5 h	[50]
	Fluorescence	*S. typhimurium*	Water, poultry packing liquid	-	100–1000 CFU /mL	5 min	[51]
	Fluorescence	Thiram	Water	-	0.1 µM	~15 min	[52]
	Fluorescence	*E. coli* O157:H7, *S. paratyphi A, S. paratyphi B, S. paratyphi C, S. typhi, S. enteritidis*, *S. choleraesuis, Vibrio cholera* O1, *V. cholera* O139, and *V. parahaemolyticus*	Dairy products, marine products, beverages, snacks, and meats	External step of homogenization	10^4^–10^5^ CFU /mL	20 min	[53]
	Fluorescence	i) Mercury (II) ion ii) Silver (I) ion iii) neomycin	Water	-	i) 121 nM, ii) 47 nM iii) 153 nM	10 min	[54]
	Fluorescence	*E. coli*	Water	On-board DNA extraction and amplification	5 cells	60 min	[55]
	Electrochemical	Nitrite	Water	External steps of extraction and filtration	0.1 µM	~1h	[56]
	Electrochemical	Ethanol	Beer	-	0.52 mM	1 h	[57]
	Electrochemical	*E. coli* and *Bacillus* sp.	Water	-	1.9 × 10^3^ CFU/mL	45 min	[58]
	Colorimetric and electrochemical	i) Lead (II) ion ii) Cadmium (II) ion iii) Copper (II) ion	Water, rice and fish samples	External step of filtration for water samples External steps of grinding, extraction and filtration for rice and fish samples	i) 0.1 ng/mL ii) 0.1 ng/mL iii) 5 ng/mL	~10 min	[59]
	Chemiluminescence	*Salmonella* sp.	Water	-	2.6 × 10^7^ CFU/mL	35 min	[60]
	Surface-enhanced Raman scattering	i) Thiram ii) Thiabendazole iii) Methyl parathion	Apples, oranges, tomatoes, and green vegetables	External steps of fruit cutting	i) 0.26 ng/cm^2^ ii) 28 ng/cm^2^ iii) 7.4 ng/cm^2^	~5 min	[61]
	Surface-enhanced Raman scattering	i) Thiram ii) Ferbam	Water	-	i) 0.46 nM ii) 0.49 nM	~5 min	[62]
Chip-based devices	Colorimetric	Aflatoxin B1	Corn	External steps of homogenization and extraction	3 ppb	1 h	[9]
	Colorimetric	i) Gluten ii) Ara h1	i) Wheat ii) Peanut	External steps of extraction and filtration	i) 4.77 ng/mL ii) 15.2 ng/mL	15–20 min	[63]
	Colorimetric	i) Lead (II) ion ii) Aluminium (III) ion	Water	-	i) 30 ppb ii) 89 ppb	8–10 min	[64]
	Colorimetric	Malathion	Apple	External steps of extraction and centrifugation	100 ppb	20 min	[65]
	Colorimetric	Tetrabromodiphenyl ether	Water	-	0.01 µg/L	12 min	[66]
	Fluorescence	i) *Staphylococcus aureus* ii) *V. parahaemolyticus*	Shrimp	External step of homogenization	i) 1000 CFU/mL ii) 1000 CFU/mL	1 h	[10]
	Fluorescence	*S. typhimurium*	Chicken extract	Undisclosed external step of extraction + on-board immunomagnetic separation of target	1000 CFU/mL	17 min	[67]
	Fluorescence	Ara h1	Biscuit	External steps of extraction and filtration	56 ng/mL	10 min	[68]
	Fluorescence	Listeria monocytogenes	Beef filtrate	External steps of homogenization and filtration + on-board immunomagnetic separation of target	10 CFU/mL	33 min	[69]
	Fluorescence	i) *E. coli* ii) *Proteus hauseri* iii) *V. parahaemolyticus* iv) *S. enterica*	Serum	-	i) 3 copies/µL ii) 3 copies/µL iii) 3 copies/µL iv) 3 copies/µL	2 h	[70]
	Fluorescence	Anti-recombinant bovine somatropin antibody	Milk	External steps of extraction and filtration	-	2.5 h	[71]
	Fluorescence	*S. enterica*	Pork meat	External step of extraction + on-board immunomagnetic separation of target and DNA extraction	10 cells/ µL	40 min	[72]
	Electrochemical	*E. coli*	Water	-	300 CFU/mL	1 min	[73]
	Electrochemical	i) *E. coli* O157:H7 ii) *S. aureus*	Phosphate buffered saline	-	i) 100 CFU/mL ii) 100 CFU/mL	30 min	[74]
	Electrochemical	*E. coli* O157:H7	Milk	External steps of dilution and immunomagnetic separation of target	12 CFU/mL	1.5 h	[75]
	Electrochemical	*S. typhimurium*	Milk	External step of immunomagnetic separation of target	7.7 cells/mL	1 h	[76]
	Electrochemical	Clenbuterol	Water	-	0.076 ng/mL	6 min	[77]
	Electrochemical	i) *E. coli* ii) *S. aureus*	Peptone water	-	i) 100 CFU/mL ii) 100 CFU/mL	1 min	[78]
	Surface plasmon resonance	Ochratoxin A	Wine and peanut oil	External step of extraction for wine External steps ofcentrifugation and filtration for peanut oil	0.005 ng/mL	2.5 min	[79]
	Surface plasmon resonance	i) *E. coli* O157:H7 ii) *Salmonella* sp.	a) Cucumber b) Hamburger	External steps of homogenization and centrifugation	i) a) 57 CFU/mL; b)17 CFU/mL ii) a) 7400 CFU/mL; b) 11,700 CFU/mL	55 min	[80]
	Surface plasmon resonance	*E. coli*	Phosphate buffered saline	-	100000 CFU/mL	20 min	[81]
	Surface plasmon resonance	i) *Lactobacillus acidophilus* ii) *S. typhimurium* iii) *P. aeruginosa*	Phosphate buffered saline	-	i) 10,000 CFU/mL ii) 10,000 CFU/mL iii) 10,000 CFU/mL	1 h	[82]
	Gas-pressure induced ink bar advancement	Bovine catalase	Milk	-	20 µg/mL	3 min	[83]
	Gas-pressure induced ink bar advancement	Aflatoxin B1	Beer	External step of degassing and filtration	1.77 nM	1.5 h	[84]
	Turbidity	*E. coli* O157:H7	Apple juice and milk	External step of DNA extraction	1 CFU/mL	1.75 h	[85]
Other devices:							
Thread-based devices	Colorimetric	*S. enterica*	i) Milk ii) Orange juice iii) Lettuce	External steps of homogenization and filtration for lettuce	i) 1000 CFU/mL ii) 1000 CFU/mL iii) 5000 CFU/mL	10 min	[86]
	Electrochemical	Phenol	Water	-	2.94 nM	-	[87]
Tube-based devices	Colorimetric	Fluoride	Water	-	0.6 ppm	1 min	[88]
	Colorimetric	Mercury (II) ion	Water	-	0.28 ng/mL	20 min	[89]
Cuvette-based devices	Colorimetric	Mercury (II) ion	Water	External step of filtration	3.5 ppb	20 min	[90]
	Colorimetric	Fluoride	Water	-	0.0256 mg/L	-	[91]
Well plate-based devices	Colorimetric	i) Tetracyclines ii) Quinolones	Milk	External step of dilution	i) 1.51 ng/mL ii) 1.74 ng/mL	42 min	[92]
	Colorimetric	i) Saxitoxin ii) Okadaic acid	Shellfish	External steps of homogenization, extraction, centrifugation and filtration	i) 0.03 ng/mL ii) 0.4 ng/mL	1 h	[93]
Disc-based device	Fluorescence	i) *E. coli* ii) *Salmonella sp.* iii) *V. cholerae*	Chicken meat	External steps of homogenization, centrifugation and DNA extraction	i) 0.03 pg/µL DNA ii) 0.03 µg/µL DNA iii) 0.03 µg/µL DNA	1 h	[94]
Glass slide-based devices	Fluorescence	Anti-recombinant bovine somatropin antibody	Milk	External steps of extraction and filtration	-	2.5 h	[95]
Nanomaterial-based devices	Fluorescence	*S. typhimurium*	Milk	-	100 CFU/mL	45 min	[96]
	Electrochemical	*E. coli* O157:H7	Water, fruit juice and milk	-	3.8 CFU/mL	-	[97]
	Gas-pressure induced ink bar advancement	i) *S. enteriditis* ii) *E. coli* O157:H7	Milk	External step of immunomagnetic separation of target	i) 10 CFU/mL ii) 10 CFU/mL	2 h	[98]
	Chemiluminescence	*S. typhimurium*	Milk	On-board immunomagnetic separation of target	10 CFU/mL	2.5 h	[99]
